# Structural Differences Across Multiple Visual Cortical Regions in the Absence of Cone Function in Congenital Achromatopsia

**DOI:** 10.3389/fnins.2021.718958

**Published:** 2021-10-14

**Authors:** Rebecca Lowndes, Barbara Molz, Lucy Warriner, Anne Herbik, Pieter B. de Best, Noa Raz, Andre Gouws, Khazar Ahmadi, Rebecca J. McLean, Irene Gottlob, Susanne Kohl, Lars Choritz, John Maguire, Martin Kanowski, Barbara Käsmann-Kellner, Ilse Wieland, Eyal Banin, Netta Levin, Michael B. Hoffmann, Antony B. Morland, Heidi A. Baseler

**Affiliations:** ^1^Department of Psychology, University of York, York, United Kingdom; ^2^York Neuroimaging Centre, Department of Psychology, University of York, York, United Kingdom; ^3^Language and Genetics Department, Max Planck Institute for Psycholinguistics, Nijmegen, Netherlands; ^4^Department of Ophthalmology, University Hospital, Otto von Guericke University, Magdeburg, Germany; ^5^MRI Unit, Department of Neurology, Hadassah Medical Center, Jerusalem, Israel; ^6^University of Leicester Ulverscroft Eye Unit, University of Leicester, Leicester Royal Infirmary, Leicester, United Kingdom; ^7^Molecular Genetics Laboratory, Institute for Ophthalmic Research, Centre for Ophthalmology, University Clinics Tübingen, Tübingen, Germany; ^8^School of Optometry and Vision Sciences, University of Bradford, Bradford, United Kingdom; ^9^Department of Neurology, University Hospital, Otto von Guericke University, Magdeburg, Germany; ^10^Department of Ophthalmology, Saarland University Hospital and Medical Faculty of the Saarland University Hospital, Homburg, Germany; ^11^Department of Molecular Genetics, Institute for Human Genetics, University Hospital, Otto von Guericke University, Magdeburg, Germany; ^12^Degenerative Diseases of the Retina Unit, Department of Ophthalmology, Hadassah Medical Center, Jerusalem, Israel; ^13^Center for Behavioral Brain Sciences, Magdeburg, Germany; ^14^York Biomedical Research Institute, University of York, York, United Kingdom; ^15^Hull York Medical School, University of York, York, United Kingdom

**Keywords:** achromatopsia, congenital visual impairment, anatomical brain regions, visual areas, structural plasticity, parallel visual pathways, ventral and dorsal pathways

## Abstract

Most individuals with congenital achromatopsia (ACHM) carry mutations that affect the retinal phototransduction pathway of cone photoreceptors, fundamental to both high acuity vision and colour perception. As the central fovea is occupied solely by cones, achromats have an absence of retinal input to the visual cortex and a small central area of blindness. Additionally, those with complete ACHM have no colour perception, and colour processing regions of the ventral cortex also lack typical chromatic signals from the cones. This study examined the cortical morphology (grey matter volume, cortical thickness, and cortical surface area) of multiple visual cortical regions in ACHM (*n* = 15) compared to normally sighted controls (*n* = 42) to determine the cortical changes that are associated with the retinal characteristics of ACHM. Surface-based morphometry was applied to T1-weighted MRI in atlas-defined early, ventral and dorsal visual regions of interest. Reduced grey matter volume in V1, V2, V3, and V4 was found in ACHM compared to controls, driven by a reduction in cortical surface area as there was no significant reduction in cortical thickness. Cortical surface area (but not thickness) was reduced in a wide range of areas (V1, V2, V3, TO1, V4, and LO1). Reduction in early visual areas with large foveal representations (V1, V2, and V3) suggests that the lack of foveal input to the visual cortex was a major driving factor in morphological changes in ACHM. However, the significant reduction in ventral area V4 coupled with the lack of difference in dorsal areas V3a and V3b suggest that deprivation of chromatic signals to visual cortex in ACHM may also contribute to changes in cortical morphology. This research shows that the congenital lack of cone input to the visual cortex can lead to widespread structural changes across multiple visual areas.

## Introduction

Congenital achromatopsia (ACHM; also known as rod monochromacy) is a largely stationary, genetically heterogeneous and predominantly autosomal recessive retinal disorder with a prevalence of approximately ∼1 in 30,000 people (Francois, 1961; Kohl et al., 2004; Aboshiha et al., 2016). Most cases are caused by mutations in one of the several genes expressed in the retinal phototransduction pathway of the cone photoreceptor. As a result, ACHM is characterised by a lack of function in all three cone photoreceptors from birth. The functional integrity of cone photoreceptors is fundamental for the mediation of photopic (bright light) vision, high visual acuity and colour perception. In the normally-sighted, the central fovea of the retina is composed exclusively of cone photoreceptors providing high visual acuity. With only functioning rods, individuals with complete ACHM have a central scotoma where rods are absent and a complete loss of colour vision from birth, along with reduced visual acuity (Haegerstrom-Portnoy et al., 1996a,b; Remmer et al., 2015; Hirji et al., 2018). In normally-sighted individuals, the foveal region of the visual field dominated by cones is overrepresented in the visual cortex (cortical magnification). Crucially, this means that in ACHM a disproportionately large area of the visual cortex receives atypical input due to the defective cone photoreceptors. Thus, it is possible that the lack of visual input to foveal representations within visual regions throughout the cortex may influence the structural characteristics of the visual cortex within this patient population.

The brain contains multiple representations of the visual field, becoming functionally specialised along the visual processing hierarchy. Beyond early occipital areas V1, V2, and V3, higher areas in the ventral stream are important for colour, pattern and shape/form processing (Zeki et al., 1991; McKeefry and Zeki, 1997; Wade et al., 2002; Goddard et al., 2011; Winawer and Witthoft, 2015), while dorsal areas are involved in the analysis of spatial characteristics such as object motion, position, depth and visually guided grasping (Ungerleider and Mishkin, 1982; Goodale and Milner, 1992). In particular, human ventral area V4 responds most strongly to chromatic stimuli (Zeki et al., 1991; Wade et al., 2002; Goddard et al., 2011), and responses in ventral occipital cortex (VO) have been correlated with the perceptual experience of colour (Jiang et al., 2007). Furthermore, damage to ventral areas such as V4 leads to a loss of colour perception (cerebral achromatopsia) (Zeki, 1990). Given the importance of cones for colour perception and high acuity vision needed for processing shape/form, it is possible that lack of cone input in ACHM might affect ventral visual areas more significantly than dorsal areas. Indeed, a behavioural study by Burton et al. (2016) supports this hypothesis, reporting that individuals with ACHM are more impaired in global form perception relative to global motion and biological motion perception.

To date, at least five genes, GNAT2, PDE6C, PDE6H, CNGA3, and CNGB3, have been identified as responsible for over 90% of congenital ACHM cases (Wissinger et al., 2001; Johnson et al., 2004; Varsányi et al., 2005; Thiadens et al., 2009; Liang et al., 2015; Zelinger et al., 2015; Sun et al., 2020). Of these, the vast majority are caused by mutations in either CNGA3 or CNGB3 genes (Kohl et al., 2004; Liang et al., 2015; Zelinger et al., 2015; Sun et al., 2020). Current clinical trials are testing gene-therapeutic interventions to treat congenital ACHM by restoring cone function in the eye (Fischer et al., 2020; Reichel et al., 2021; NCT03758404, NCT03001310, NCT03278873, NCT02935517, NCT02599922, and NCT02610582).

The consequences of potential cortical changes in ACHM on efforts to restore vision are currently unknown. However, we can draw on changes to the posterior visual pathway that have been documented in other visual deficits to provide some context (Prins et al., 2016). Previous research has reported significant structural changes in visual processing pathways in the brain both in individuals with congenital blindness (Ptito et al., 2008; Jiang et al., 2009; Park et al., 2009; Aguirre et al., 2016; Bridge and Watkins, 2019) and acquired defects (Boucard et al., 2009; Hernowo et al., 2014; Brown et al., 2016; Prins et al., 2016; Hanson et al., 2019). Functional changes have been identified in another ophthalmological disorder, i.e., amblyopia, in higher visual areas including V4 and MT+ (Wong, 2012). Structural changes have also been reported in totally blind individuals specifically in connectivity to the ventral visual areas (Reislev et al., 2016a,b). In models of glaucoma, it is evident that the posterior visual pathway undergoes degeneration, which would likely prevent full restoration of vision (Yucel and Gupta, 2015). If cortical atrophy is detected in ACHM, it is possible that degeneration has occurred and could limit restoration of vision.

Efforts to restore vision in different disorders have been cautiously optimistic, but have produced mixed results. In late-onset disorders such as age-related cataract, visual acuity immediately improves following corrective surgery, and this is associated with an increase in grey matter volume of visual brain areas (Lou et al., 2013; Lin et al., 2018). Similarly, in late-blind participants with retinitis pigmentosa, motion detection and BOLD responses to visual stimuli were enhanced following implantation of a retinal prosthesis even after years of deprivation (Castaldi et al., 2016). However, visual recovery evident in these studies may have been strictly dependent on the presence of early visual experience during childhood (Hadad et al., 2015). In early-onset disorders, such visual experience is absent. Even when the optical image has been fully restored following surgery, vision is not always restored to normal. In cases of early blindness due to corneal opacity, after corneal replacement participants still perform poorly on higher level visual tasks requiring shape, object and face processing (Fine et al., 2003) or colour discrimination (Ackroyd et al., 1974). Following surgery to correct congenital cataract, the degree of vision restoration is inversely related to the extent of changes in visual cortex (Guerreiro et al., 2015, 2016). Overall, these studies suggest that restoring vision to normal must involve normal cortical function and that anatomical biomarkers could provide a valuable indicator of the extent to which function may return to normal, particularly in the case of a congenital deficit.

Using gene therapeutic interventions, such as those well established in Leber’s Congenital Amaurosis, visual and behavioural outcomes have also been variable (for review see: Chiu et al., 2021). However, evidence that cortical plasticity is still possible later in life was provided by an fMRI study reporting increased responses in visual cortex following gene therapy when participants with Leber’s Congenital Amaurosis were treated in adulthood (Mowad et al., 2020). In ACHM, early studies also report promising but variable results following gene therapy (Farahbakhsh et al., 2020; Fischer et al., 2020; Reichel et al., 2021). In a study of two adult ACHM participants, minor improvements in visual acuity and a reduction in levels of photoaversion were found following treatment (McKyton et al., 2021). Furthermore, population receptive field sizes were reduced in early visual areas following treatment, suggesting that some restoration of cortical function is possible. However, although participants were now able to detect differences in the red end of the spectrum, there was no improvement in colour discrimination nor were fMRI responses detectable in colour-specific brain areas. The absence of improved colour discrimination could be due to insufficient restoration of retinal function or due to limitations in visual cortex. Nevertheless, a lack of colour responses in extrastriate brain areas along with an inability to discriminate colours suggest that long-term deficits may persist.

The aim of the current study was to evaluate how the deprivation of foveal and chromatic vision in participants with ACHM affect the development and structural integrity of multiple visual regions in striate and extrastriate cortex. Given the absence of functioning cones in ACHM, we hypothesised that ventral visual areas would be more affected than dorsal areas. We found that early visual areas V1 to V3 exhibit atrophic changes in ACHM. Similar changes were also present in ventral region V4, but not in dorsal areas V3a and V3b at an equivalent level in the visual hierarchy. We provide evidence therefore that the structural development of visual brain areas driven predominantly by cone input is particularly affected in ACHM.

## Materials and Methods

### Participants

High resolution structural scans were collected at three sites from 42 control participants with normal or corrected to normal vision (mean age ± SD: 30.29 ± 9.72 years; 19 males) and 15 participants with both genetically confirmed ACHM (biallelic CNGA3 (*n* = 10) or CNGB3 (*n* = 5) mutations; see Table 1) and electroretinographically confirmed absence of cone function (mean age ± SD: 36.73 ± 10.95 years; 9 males). An independent samples *t*-test between groups found no significant age difference between groups [*t*(55) = −2.02. *p* = 0.056]. However, given the difference in mean age was close to the *p* < 0.05 cut-off, a subsequent analysis assessed age as a possible confound, along with scanner site, gender, and global metrics (see section “Results”).

**TABLE 1:** Summary of patient demographics showing participant group (ACHM, participants with congenital achromatopsia; C, control participants), gender (m, male; f, female), age, scanner site (HMC, Hadassah Medical Centre; UM, University of Magdeburg; UY, University of York), and genotype.

**Participant**	**Gender**	**Age**	**Site**	**Genotype**
ACHM	m	34	HMC	*CNGA3*
ACHM	m	41	HMC	*CNGA3*
ACHM	m	35	HMC	*CNGA3*
ACHM	f	41	HMC	*CNGA3*
ACHM	f	42	HMC	*CNGA3*
ACHM	m	28	HMC	*CNGA3*
ACHM	m	18	UM	*CNGB3*
ACHM	f	55	UM	*CNGA3*
ACHM	f	29	UM	*CNGB3*
ACHM	m	45	UM	*CNGB3*
ACHM	m	22	UM	*CNGA3*
ACHM	f	40	UY	*CNGB3*
ACHM	m	28	UY	*CNGB3*
ACHM	m	34	UY	*CNGA3*
ACHM	f	51	UY	*CNGA3*
C	m	25	HMC	–
C	f	33	HMC	–
C	m	19	HMC	–
C	f	22	HMC	–
C	f	24	HMC	–
C	m	34	HMC	–
C	f	27	HMC	–
C	m	26	HMC	–
C	m	29	HMC	–
C	f	29	HMC	–
C	f	24	HMC	–
C	f	32	HMC	–
C	f	46	HMC	–
C	f	30	HMC	–
C	f	22	HMC	–
C	f	57	HMC	–
C	f	23	HMC	–
C	m	23	HMC	–
C	m	50	HMC	–
C	m	43	HMC	–
C	f	25	HMC	–
C	f	27	HMC	–
C	m	26	HMC	–
C	m	25	HMC	–
C	m	33	UM	–
C	f	58	UM	–
C	m	29	UM	–
C	m	27	UM	–
C	f	32	UM	–
C	f	53	UM	–
C	f	35	UM	–
C	m	27	UM	–
C	f	26	UY	–
C	f	26	UY	–
C	m	35	UY	–
C	m	29	UY	–
C	f	23	UY	–
C	m	24	UY	–
C	f	30	UY	–
C	m	23	UY	–
C	f	19	UY	–
C	m	22	UY	–

Experimental protocols received approval from the site-specific ethics committees and were in accordance with the Declaration of Helsinki.

### MRI Protocol

#### University of York (10 Controls, 4 Achromatopsia)

A single, high resolution, anatomical, T1-weighted scan (TR, 2500 ms; TE, 2.26ms; TI, 900 ms; voxel size, 1 × 1 × 1 mm^3^; flip angle, 7°; matrix size, 256 × 256 × 176, total acquisition time, 306 s) was acquired on each participant using a 64-channel head coil on a SIEMENS MAGNETOM Prisma 3T scanner at the York Neuroimaging Centre (YNiC).

#### Hadassah Medical Centre (24 Controls, 6 Achromatopsia)

A single, high resolution, anatomical, T1-weighted scan (TR, 2300 ms; TE, 1.5 ms; TI, 900 ms; voxel size, 1 × 1 × 1 mm^3^; flip angle, 9°; matrix size, 256 × 256 × 160, total acquisition time, 278 s) was acquired on each participant using a 32-channel head coil on a SIEMENS MAGNETOM Skyra 3T scanner at the Edmond & Lily Safra Centre for Brain Sciences, Hebrew University of Jerusalem.

#### University of Magdeburg (8 Controls, 5 Achromatopsia)

A single, high resolution, anatomical, T1-weighted scan (TR, 2500 ms; TE, 2.82 ms; TI, 1100 ms; voxel size, 1 × 1 × 1 mm^3^; flip angle, 7°; matrix size, 256 × 256 × 192, total acquisition time, 560 s) was acquired on each participant using a 64-channel head coil on a SIEMENS MAGNETOM Prisma 3T scanner at the University Hospital, Magdeburg, Germany.

### Data Pre-processing

Cortical reconstruction and volumetric segmentation of the T1-weighted scans and surface-based morphology analysis were performed using the Freesurfer analysis suite Version 6.0 (Dale et al., 1999; Fischl et al., 1999). This included the removal of non-brain tissue (Ségonne et al., 2004), automated Talairach transformation, intensity normalisation (Sled et al., 1998), tessellation of the grey/white matter and pial boundaries (grey/cerebrospinal fluid) with automated topology correction and surface deformation (Dale et al., 1999; Fischl et al., 1999; Ségonne et al., 2007). Subsequently, the cortical surface was inflated and registered to a sphere (Fischl et al., 1999) and the surface parcellated according to gyral and sulcal structures (Ségonne et al., 2004; Desikan et al., 2006).

The final surface reconstruction was inspected for potential cortical segmentation errors (for example areas where dura mater was incorrectly included in the grey matter surface during the initial automated segmentation) and, when necessary, manually corrected using the FreeView Visualisation GUI. Manual editing was split between two expert observers who were blind to participant identity and group to avoid bias. Minor edits to the pial surface were made in 50% of ACHM and 50% of control participants. Edits were primarily of the skull and dura mater located at parietal, motor and frontal cortical regions with only a small minority of participants requiring edits in the occipital cortex. All manually corrected reconstructions were rerun (‘autoreconall2’) utilising the edited brainmask.mgz files.

### Data Analysis

A subsequent region-of-interest (ROI)-based analysis was applied where we compared differences in three surface-based measures between ACHM and their demographically matched controls: mean cortical volume (mm^3^), cortical thickness (mm) and surface area (mm^2^).

Cortical volume was computed as described in Winkler et al. (2018). Briefly, three vertices defining a face in the white surface and three matching vertices in the pial surface form an oblique truncated triangular pyramid; the volumes of these are subsequently computed and summed together for the whole ROI. Cortical thickness was defined as the shortest distance between each grey/white matter boundary vertex and the pial surface (grey matter/cerebrospinal fluid boundary) and vice versa. The final value depicted the average of the two thickness values measured, and thickness values were then averaged across each ROI (Fischl and Dale, 2000). Surface area was measured by calculating the summed surface area across each ROI of each triangle of the surface mesh, the unit used to connect the cortical surface between each vertex.

**FIGURE 1:**
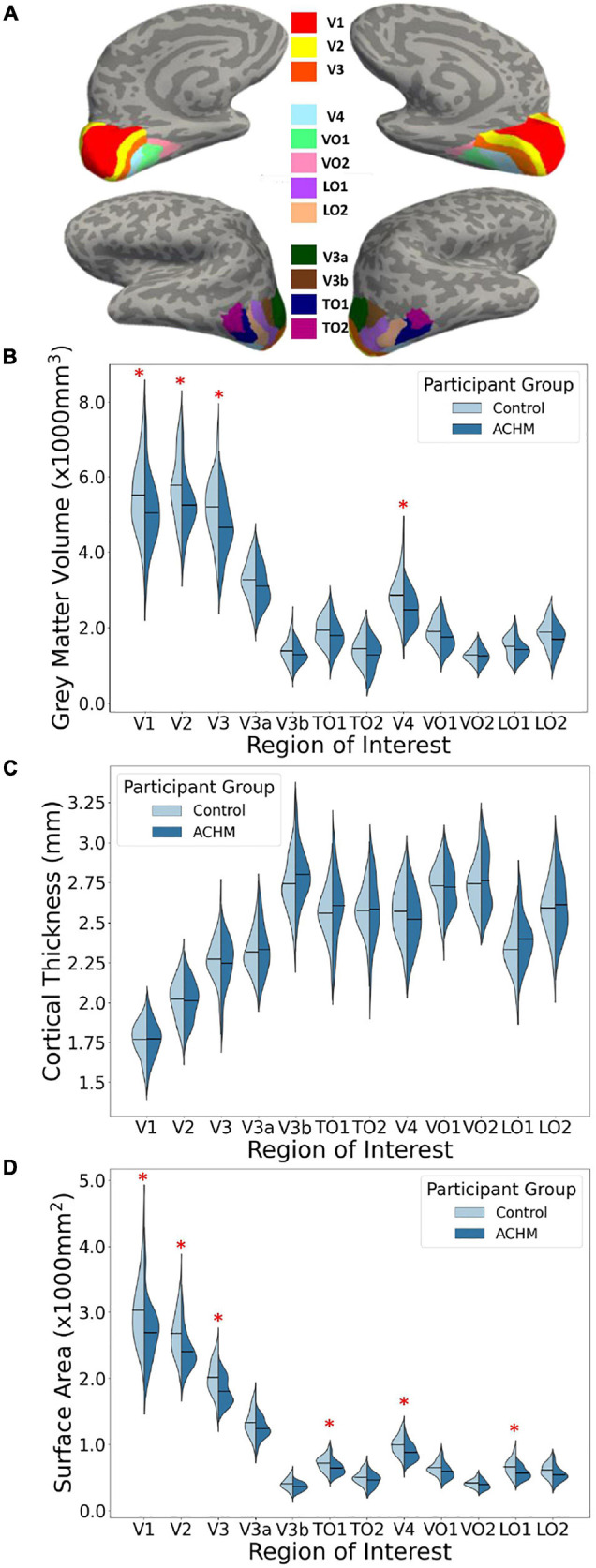
Regions of interest and mean grey matter volume, cortical thickness and surface area for ACHM and controls. **(A)** An inflated surface of one control participant showing the cortical surface of the atlas-defined brain areas used in analysis (Benson et al., 2014; Benson and Winawer, 2018; https://osf.io/knb5g/). **(B)** A violin plot showing the distribution of mean grey matter volume averaged across the two hemispheres of controls and ACHM for each ROI. Horizontal lines within the violins indicate means for each group. Red stars indicate significant differences between groups in *post hoc* comparisons (*p* < 0.05). **(C)** A violin plot showing the distribution of mean cortical thickness averaged across the two hemispheres of controls and ACHM for each ROI. Details as in **(B)**. **(D)** A violin plot showing the distribution of mean cortical surface area averaged across the two hemispheres of controls and ACHM for each ROI. Details as in **(B)**. Red stars indicate significant differences between groups in *post hoc* comparisons (*p* < 0.05).

Regions-of-interest used for this analysis stream were derived using the anatomically defined retinotopy atlas (Benson et al., 2014) implemented in the python analysis toolbox ‘neuropythy’ (Benson and Winawer, 2018). The atlas then predicted several Freesurfer-based maps (visual area, eccentricity, polar angle, and pRF size), which were used to delineate twelve ROI labels for each participant. The ROIs encompass the entire cortical field representations of areas V1, V2, V3, V3a, V3b, TO1, TO2, V4, VO1, VO2, LO1, and LO2 (Figure 1A).

## Results

First, the data were evaluated from each hemisphere separately. A 2 × 2 × 12 mixed measures ANOVA was performed with between-subjects factor participant group (2 levels – ACHM and controls), and within-subjects factors of hemisphere (2 levels – left and right) and ROI (12 levels). Huynh-Feldt correction was applied to correct for sphericity of the data. There was no significant interaction between participant group, hemisphere, and ROI in any of the three metrics (Supplementary Table 1). Since hemisphere will have no effect on any interaction found between participant group and ROI, the data were combined across hemispheres. Extracted values of surface area and volume were averaged for each ROI and for each participant. For cortical thickness, the values were weighted by the respective surface area value and the mean cortical thickness. Values were derived by summing the product of the thickness and surface areas on the right and left hemispheres, respectively, and dividing this by the sum of the right and left surface area.


(1) $$\frac{\begin{array}{c} \left(lh.Thickness\times lh.SurfaceArea\right)+(rh.Thickness\\ \times rh.SurfaceArea) \end{array}}{l\,h.S\,u\,r\,f\,a\,c\,e\,A\,r\,e\,a+r\,h.S\,u\,r\,f\,a\,c\,e\,A\,r\,e\,a}$$

Results of the data averaged across hemispheres are shown in Figures 1B–D. To determine whether the effects of age, scanner site, gender, and global brain metrics influenced any of the three outcome measures, these variables were entered as potential confounds using analysis of covariance (ANCOVA). Repeated measures ANCOVAs were performed for each measurement type (grey matter volume, cortical thickness, and cortical surface area) including the two main variables of interest: participant group (2 levels) and ROI (12 levels). Huynh-Feldt correction was applied to correct for sphericity of the data.

There was no significant main effect of age, gender, scanner site or global brain metrics, and no interactions between any of these variables and participant group. The only significant interaction between the potential confounds and variables of interest was in grey matter volume for ROI × scanner site × gender [*F*(8.44, 181.56) = 2.64, *p* = 0.008]. This may reflect the differing number of each gender at each scanner site. All potential confounds are nevertheless accounted for in the remaining results (see Table 2).

**TABLE 2:** Results of analysis of covariance to assess effects of possible confounds age, scanner site, gender and global metrics.

**Source**	**df1, df2**	***F***	***p***	**Effect size**
**Grey matter volume**				
Participant group (A)	1, 43	4.87	**0.033**	0.10
ROI (B)	4.22, 181.56	3.63	**0.006**	0.08
Scanner site (C)	2, 43	0.61	0.549	0.03
Age (D)	1, 43	1.10	0.299	0.03
Gender (E)	1, 43	0.30	0.587	0.01
Global volume (F)	1, 43	0.95	0.335	0.02
A × B	4.22, 181.56	4.85	**0.001**	0.10
A × C	2, 43	0.02	0.976	0.00
A × E	1, 43	0.00	0.963	0.00
A × B × C	8.44, 181.56	0.39	0.934	0.02
A × B × E	4.22, 181.56	0.86	0.492	0.02
A × C × E	2, 43	0.08	0.920	0.00
A × B × C × E	8.44, 181.56	0.30	0.968	0.01
B × C	8.44, 181.56	1.19	0.308	0.05
B × D	4.22, 181.56	2.14	0.074	0.05
B × E	4.22, 181.56	0.53	0.723	0.01
B × F	4.22, 181.56	0.48	0.764	0.01
B × C × E	8.44, 181.56	2.64	**0.008**	0.11
C × E	2, 43	0.77	0.471	0.03
**Cortical thickness**				
Participant group (A)	1, 43	0.00	0.962	0.00
ROI (B)	11.00, 473.00	1.11	0.352	0.03
Scanner site (C)	2, 43	0.86	0.432	0.04
Age (D)	1, 43	0.03	0.861	0.00
Gender (E)	1, 43	0.20	0.658	0.01
Global thickness (F)	1, 43	0.51	0.478	0.01
A × B	11.00, 473.00	1.55	0.112	0.04
A × C	2, 43	0.66	0.522	0.03
A × E	1, 43	0.00	0.948	0.00
A × B × C	22.00, 473.00	0.85	0.666	0.04
A × B × E	11.00, 473.00	0.61	0.823	0.01
A × C × E	2, 43	2.94	0.064	0.12
A × B × C × E	22.00, 473.00	0.68	0.862	0.03
B × C	22.00, 473.00	0.79	0.746	0.04
B × D	11.00, 473.00	0.65	0.783	0.02
B × E	11.00, 473.00	0.54	0.875	0.01
B × F	11.00, 473.00	0.86	0.582	0.02
B × C × E	22.00, 473.00	0.50	0.975	0.02
C × E	2, 43	3.68	**0.033**	0.15
**Cortical surface area**				
Participant group (A)	1, 43	7.52	**0.009**	0.15
ROI (B)	2.26, 97.28	2.72	0.064	0.06
Scanner site (C)	2, 43	0.34	0.712	0.02
Age (D)	1, 43	1.79	0.188	0.04
Gender (E)	1, 43	0.20	0.656	0.01
Global surface area (F)	1, 43	0.71	0.403	0.02
A × B	2.26, 97.28	6.62	**0.001**	0.13
A × C	2, 43	0.03	0.968	0.00
A × E	1, 43	0.10	0.749	0.00
A × B × C	4.53, 97.28	0.10	0.989	0.01
A × B × E	2.26, 97.28	0.81	0.459	0.02
A × C × E	2, 43	0.62	0.542	0.03
A × B × C × E	4.53, 97.28	0.46	0.788	0.02
B × C	4.53, 97.28	0.71	0.601	0.03
B × D	2.26, 97.28	2.89	0.054	0.06
B × E	2.26, 97.28	0.05	0.967	0.00
B × F	2.26, 97.28	0.61	0.565	0.01
B × C × E	4.53, 97.28	2.35	0.052	0.10
C × E	2, 43	0.27	0.765	0.01

*Effect size shown is partial eta-squared. Huynh-Feldt correction applied to correct for violation of sphericity. Significance is illustrated by boldface.*

The main effect of participant group was significant for grey matter volume [*F*(1,43) = 4.87, *p* = 0.033] and surface area [*F*(1,43) = 7.52, *p* = 0.009] but was not significant for cortical thickness [*F*(1,43) < 0.01, *p* = 0.962]. This indicates that mean volume and surface area are reduced overall in all visual areas tested here in ACHM compared to controls, as seen in Figure 1. The main effect of ROI was significant for grey matter volume [*F*(4.22, 181.56) = 3.63, *p* = 0.006] but not for cortical thickness [*F*(11, 473) = 1.11, *p* = 0.352] or surface area [*F*(2.26, 97.28) = 0.064]. This is expected, as differences in visual area size are well documented in the literature (Wandell et al., 2007). Critically, however, the interaction between ROI and participant group was also significant for volume [*F*(4.22, 181.56) = 4.85, *p* = 0.001] and surface area [*F*(2.26, 97.28) = 6.62, *p* = 0.001], but was not significant for cortical thickness [*F*(11,473) = 1.55, *p* = 0.112]. Considering these interactions, we performed *post hoc* pairwise comparisons to determine if there was a significant difference between ACHM participants and controls in grey matter volume and surface area for each of the 12 ROIs, adjusting for multiple comparisons using Bonferroni correction (Table 3). This analysis revealed four areas which showed a significant difference between ACHM and controls for grey matter volume (V1: *p* = 0.006, V2: *p* = 0.019, V3: *p* = 0.024, V4, *p* = 0.029) and six areas for surface area (V1: *p* = 0.008, V2: *p* = 0.004, V3: *p* = 0.007, V4: *p* = 0.0333, LO1: *p* = 0.015, TO1: *p* = 0.046).

**TABLE 3:** *Post hoc* between group comparisons following analysis of variance in Table 2, Bonferroni-corrected.

**Area**	**Grey matter volume** ***p*-Value**	**Surface area** ***p*-Value**
V1	**0.006**	**0.008**
V2	**0.019**	**0.004**
V3	**0.024**	**0.007**
V3a	0.317	0.091
V3b	0.339	0.175
TO1	0.438	**0.046**
TO2	0.117	0.101
V4	**0.029**	**0.033**
VO1	0.297	0.224
VO2	0.939	0.580
LO1	0.170	**0.015**
LO2	0.156	0.076

*Significance is illustrated by boldface.*

## Discussion

The aim of this study was to investigate any potential morphological changes in visual regions of the brain in participants with ACHM compared to normally sighted controls. Significant reductions were found in ACHM relative to controls for both volume and surface area, but not for thickness. Decreased volume appears to be driven largely by reductions in surface area rather than thickness. Our results reveal widespread morphological alterations throughout the visual cortex, consistent with previous neuroimaging studies of congenitally blind adults (Ptito et al., 2008; Aguirre et al., 2016; Bridge and Watkins, 2019). It appears that ventral visual area V4 is disproportionately affected by reductions in surface area and volume compared to the dorsal pathway areas V3a and V3b. Higher visual areas LO1 and TO1 also are reduced in surface area in ACHM, although not enough to drive differences in volume measurements.

### Early Visual Area Results

All early visual areas (V1, V2, and V3) showed significant decreases in grey matter volume and surface area for ACHM. These areas all have particularly large foveal representations (Wandell et al., 2007), suggesting that the substantial reduction in input to these regions in ACHM may explain the decrease in size. All of these areas also process colour information (Mullen et al., 2007; Railo et al., 2012; Hurme et al., 2020), which could contribute further reductions in these areas. In a separate study, we have extended our analysis to investigate more specifically the eccentricity dependence of structural changes within primary visual cortex and shows that cortical changes are most pronounced in central visual field representations (manuscript submitted and under review; preprint available under Molz et al., 2021).

### V4 and V3a/b Differences

Ventral area V4 was significantly lower in both surface area and volume in ACHM compared to controls. No such difference is found in dorsal areas V3a and V3b, however. This may be driven by the reduction in foveal input in ACHM, which is largely dominated by cone photoreceptors in normally-sighted adults. Previous research has demonstrated a preferential response bias to stimuli from the central visual field in ventral visual areas including V4, VO-1, and VO-2 (Brewer et al., 2005; Arcaro et al., 2009; Winawer and Witthoft, 2015). In contrast, dorsal areas appear to have an increasingly peripheral bias as one moves up the visual processing hierarchy away from primary visual cortex (Tootell et al., 1997; Wandell et al., 2007; Fattori et al., 2009). Areas with a more peripheral bias should therefore be less affected by ACHM, since peripheral vision is relatively preserved in these participants and rod function is intact. Our results showing no significant differences in ACHM in dorsal areas V3a and V3b support this hypothesis.

Ventral areas such as V4 have also been associated with chromatic vision (Bartels and Zeki, 2000; Wade et al., 2002; Brewer et al., 2005; Mullen et al., 2007, 2015; Bannert and Bartels, 2018), while dorsal regions V3a and V3b have been more aligned with motion processing (Tootell et al., 1997; Wandell et al., 2007; Fattori et al., 2009). Therefore, both the lack of chromatic signals and the reduction of foveal inputs caused by the absence of functional cones are likely to contribute to differences observed in V4 (but not V3a or V3b) in ACHM.

### Higher Visual Areas

LO1 was also significantly reduced in surface area in ACHM participants. This area is commonly associated with processing of shape and object recognition (Malach et al., 1995; Larsson and Heeger, 2006; Silson et al., 2013) a skill assisted by chromatic vision (Bramão et al., 2011, 2016). LO1 also exhibits a foveal bias (Larsson and Heeger, 2006). Therefore, the cortical deprivation thought to cause this morphological difference may be related to both the lack of foveal and chromatic input to this area.

TO1 also shows a lower surface area in ACHM. This area is part of human area MT+, is most commonly associated with visual motion processing, and has a large foveal representation (Amano et al., 2009). Individuals with ACHM often report problems with motion perception, which is generally impaired when mediated by rods compared to cones (Gegenfurtner et al., 1999), possibly due to lower temporal resolution of the rods (Hess and Nordby, 1986). Thus, both reduced foveal inputs as well as impaired motion processing may explain differences in TO1 in ACHM. This is in contrast to TO2, which has a greater emphasis of the peripheral visual field (Amano et al., 2009) and did not differ significantly between groups.

Surprisingly, ventral areas VO1 and VO2 do not show significant differences in any metric, an unexpected result as both have been associated with chromatic vision (Brewer et al., 2005; Jiang et al., 2007; Arcaro et al., 2009) and have large foveal representations (Brewer et al., 2005). It is unexpected that we failed to detect group differences in VO1 and VO2, which likely receive predominant cone input because of their role in colour processing. Unlike larger areas V1, V2, V3, V3a/b, and V4, smaller regions such as VO1 and VO2 are more likely to be prone to type two errors, which might explain the lack of sensitivity in revealing differences here, although we did detect differences in similarly sized areas such as LO1 and TO1. However, research has suggested that it is more difficult to map areas along the ventral surface accurately due to potential vessel artifacts (the ‘venous eclipse’), which might have introduced some uncertainty to area boundaries (Winawer et al., 2010; Benson and Winawer, 2018). Such factors could have contributed to the null result we found in small ventral areas such as VO1 and VO2 using an atlas-based approach.

### Cortical Thickness

We found no significant differences in cortical thickness in ACHM in any visual area. This is in contrast to increased cortical thickness reported in primary visual cortex in participants with total congenital blindness (Jiang et al., 2009; Park et al., 2009; Aguirre et al., 2016; Bridge and Watkins, 2019). However, it is important to note that participants with ACHM are still sighted, with an area of absolute blindness restricted only to the central fovea. Our prediction therefore would be that cortical thickening would be observed only in representations of the central visual field. Indeed, our preliminary analysis has found increased cortical thickness in the most central representations of primary visual cortex in ACHM (Molz et al., 2021). It remains to be seen if a total absence of input from the central fovea results in thickening of the foveal representation within higher visual areas.

## Conclusion

In summary, this study provides an overview of the structural changes present in visual cortex in ACHM compared to normally-sighted controls. This study has revealed widespread reduction in the surface area and volume of many visual areas. Differences are found particularly in areas that typically have large representations of the fovea and areas associated with chromatic vision, suggesting that both characteristics of cone vision that are absent in ACHM can affect brain morphology. It is important to remember drawing conclusions from this data that atlas-defined ROIs are based on neurotypical individuals. Therefore, when applying an atlas to brains that may differ structurally there may be limitations on the precision of defining the ROIs. However, this technique has been used successfully in the past and there does appear to be some specificity in the ROIs where group differences are found (Norman and Thaler, 2019). Also, there is evidence that topographical organisation of visual cortex follows retinotopic principles, even in congenitally blind individuals (Striem-Amit et al., 2015). An atlas-based approach can therefore be effective in identifying differences between groups, particularly when comparing with a neurotypical control group.

Structural differences in visual cortex in ACHM are important to consider when planning treatment, such as gene therapy to restore cone function to the eye. By adulthood, it is clear that deprivation of chromatic and foveal information has resulted in cortical remodelling, and it is difficult to establish from the literature whether this will limit the success of treatment or if sufficient plasticity remains into adulthood to permit the restoration of function. Research has shown that the volume and surface area of primary visual cortex mature earlier than other brain areas (Leuba and Kraftsik, 1994), completed by the end of the first postnatal year. Less is known about the rate of development of higher cortical areas, or whether structural changes in these areas will affect their ability to perform specialised visual functions. Given the rapid maturation of primary visual cortex, however, it seems advisable to apply any therapeutic interventions as early as possible.

## Data Availability Statement

The raw data supporting the conclusions of this article will be made available by the authors, following appropriate data protection guidelines.

## Ethics Statement

The study was in accordance with the Declaration of Helsinki, and was reviewed and approved by each of the site-specific ethics committees: the York Neuroimaging Centre Research Ethics Committee (York), the Hadassah Hebrew University Medical Centre Ethics Committee (Jerusalem), and the Ethical Committee of the Otto von Guericke University Magdeburg, Germany (Magdeburg). The participants provided their written informed consent to participate in this study.

## Author Contributions

HB, NL, MH, AM, and BM conceived and designed the experiments. BM, PB, AH, RL, NL, NR, AG, KA, RM, IG, SK, LC, JM, MK, BK-K, IW, and EB data acquisition. BM, AH, and PB performed the experiments. RL, LW, and BM analysed the data. RL and LW wrote the paper. HB, RL, LW, BM, AM, MH, PB, AH, NL, NR, AG, KA, RM, IG, SK, LC, JM, MK, BK-K, IW, and EB revised the manuscript. All authors contributed to the article and approved the submitted version.

## Conflict of Interest

The authors declare that the research was conducted in the absence of any commercial or financial relationships that could be construed as a potential conflict of interest.

## Publisher’s Note

All claims expressed in this article are solely those of the authors and do not necessarily represent those of their affiliated organizations, or those of the publisher, the editors and the reviewers. Any product that may be evaluated in this article, or claim that may be made by its manufacturer, is not guaranteed or endorsed by the publisher.
