# Supplementary material for: Structural Differences Across Multiple Visual Cortical Regions in the Absence of Cone Function in Congenital Achromatopsia
Source: Front Neurosci. 2021 Oct 14;15:718958. doi: 10.3389/fnins.2021.718958 (PMC8551799; doi:10.3389/fnins.2021.718958)
Supplement: Supplementary file 1 [file Table_1.docx]

**Table S1.**  Results of Analysis of Variance including separate left and right hemispheres. Huynh-Feldt correction applied to correct for violation of sphericity*.* Significance is illustrated by boldface.

| Source | df1,df2 | F | *p* | Effect Size |
| --- | --- | --- | --- | --- |
| Grey Matter Volume |  |  |  |  |
| ROI (A) | 2.95,162.03 | 841.76 | **<.001** | 0.94 |
| Participant Group (B) | 1,55 | 4.49 | **.039** | 0.08 |
| Hemisphere (C) | 1,55 | 48.45 | **<.001** | 0.47 |
| A x B | 2.95,162.03 | 2.69 | .054 | .05 |
| A x C | 7.55,415.36 | 37.42 | **<.001** | 0.41 |
| B x C | 1,55 | 0.20 | .655 | 0.004 |
| A x B x C | 7.55,415.36 | 0.46 | .847 | 0.008 |
| Cortical Thickness |  |  |  |  |
| ROI (A) | 9.15,503.33 | 411.90 | **<.001** | 0.88 |
| Participant Group (B) | 1,55 | 0.13 | .725 | 0.002 |
| Hemisphere (C) | 1,55 | 55.41 | **<.001** | 0.50 |
| A x B | 9.15,503.33 | 1.29 | .247 | 0.02 |
| A x C | 9.11,501.29 | 7.59 | **<.001** | 0.12 |
| B x C | 1,55 | 2.17 | .142 | 0.04 |
| A x B x C | 9.11,501.29 | 0.70 | .712 | 0.01 |
| Cortical Surface Area |  |  |  |  |
| ROI (A) | 1.66,91.45 | 1364.24 | **<.001** | 0.96 |
| Participant Group (B) | 1,55 | 7.67 | **.008** | 0.12 |
| Hemisphere (C) | 1,55 | 27.34 | **<.001** | 0.33 |
| A x B | 1.66,91.45 | 4.46 | **.020** | 0.08 |
| A x C | 5.05,277.53 | 45.09 | **<.001** | 0.45 |
| B x C | 1,55 | 0.24 | .626 | 0.004 |
| A x B x C | 5.05,277.53 | 0.60 | .702 | 0.01 |
